# The Prognostic Role of Glasgow Prognostic Score and C-reactive Protein to Albumin Ratio for Sarcoma: A System Review and Meta-Analysis

**DOI:** 10.1155/2020/8736509

**Published:** 2020-01-07

**Authors:** Erhu Fang, Xiaolin Wang, Jiexiong Feng, Xiang Zhao

**Affiliations:** Department of Pediatric Surgery, Tongji Hospital, Tongji Medical College, Huazhong University of Science and Technology, Wuhan 430030, China

## Abstract

**Backgrounds:**

Both pretreatment serum CRP (C-reactive protein) level and ALB (albumin) level have been found to be predictive of survival for multiple malignancies including sarcoma. Since both of the GPS (Glasgow prognostic score) and CAR (C-reactive protein to albumin ratio) are based on the combination of CRP and ALB, we conducted a meta-analysis to evaluate the prognostic role of these two parameters for sarcoma patients.

**Methods:**

A detailed literature search was conducted in MEDLINE, Embase, and Cochrane Library for relevant research publications written in English. Patients' clinical characteristics, outcomes of overall survival (OS), disease-specific survival (DSS), and disease-free survival (DFS) were extracted. Pooled hazard ratios (HRs) and corresponding 95% confidence intervals (CIs) were combined to evaluate the prognostic role of GPS or CAR.

**Results:**

Twelve articles containing 2695 patients were identified as eligible studies. The results showed that an elevated GPS was significantly correlated with poor OS (HR = 2.42; 95% CI: 1.98-2.94; *p* < 0.001; fixed-effects model), DSS (HR = 2.28; 95% CI: 1.75-2.97; *p* < 0.001; fixed-effects model), and DFS (HR = 2.05; 95% CI: 1.62-2.60; *p* < 0.001; fixed-effects model). A higher CAR also was shown to be significantly correlated with poor OS (HR = 2.23; 95% CI: 1.70-2.92; *p* < 0.001; fixed-effects model) and DFS (HR = 1.81; 95% CI: 1.7-2.58; *p* = 0.001; fixed-effects model).

**Conclusion:**

An elevated GPS is predictive of poor survival in patients with sarcomas and is promising to be used as a factor for risk stratification. A higher CAR value is also predictive of poor survival; however, the optimal CAR cut-off value is still to be determined.

## 1. Introduction

Sarcoma represents a heterogeneous group of mesenchymal malignancies that arise from soft tissue or bone, with diverse subtypes and varying degrees of aggressiveness [1, 2]. It accounts for nearly 21% of all paediatric solid malignancies and 1% of all adult solid malignancies [2, 3]. Traditional prognostic factors such as pathological grade, tumor size, tumor depth, or surgical margins have been used for risk stratification, but fail to accurately predict disease recurrence or survival [4]. About 50% of sarcoma patients with adequate local control develop distant metastases [4]. Even with the recent development of several novel chemotherapeutics, the prognosis of metastatic sarcoma remains poor [3]. New parameters are still needed to further improve the risk stratification of sarcoma.

Increasing evidence has revealed that cancer-associated systemic inflammation and malnutrition can affect the prognosis of patients with malignancies [5–8]. It is difficult to separate inflammation and nutrition status clearly. Inflammation impairs nutritional status by decreasing food intake and impairing micronutrient absorption, and malnutrition also increases the risk and severity of inflammation [9, 10].

The inflammation marker CRP (C-reactive protein) and nutrition marker ALB (albumin) are widely used and cheap clinical parameters. Both of them have been found to be predictive of survival in cancer patients [11–14]. The Glasgow prognostic score (GPS), which was developed based on the combination of CRP and ALB, is considered to reflect both inflammation and nutrition status [15–17]. Recently, numerous studies have revealed that a high level of the GPS is significantly correlated with poor survival outcome in multiple types of cancers [18–25]. Other than the GPS, the CAR (C-reactive protein to albumin ratio) is another parameter based on CRP and ALB. Many researches have also found that a high CRP/ALB ratio is an independent prognostic marker for cancer patients [26–29].

The prognostic significance of GPS and CAR has also been investigated in sarcoma patients, and controversial results have also been reported [30–42]. To our knowledge, no system review or meta-analysis has been conducted on this subject for sarcoma. In the present study, we conducted a meta-analysis and combined the results of relevant studies to evaluate the prognostic role of GPS and CAR in patients with sarcoma. The results showed that both the elevated GPS and higher CAR are significantly correlated with poor survival in sarcoma patients separately.

## 2. Materials and Methods

### 2.1. Search Strategy

The search was conducted according to the Preferred Reporting Items for Systematic Reviews and Meta-Analyses (PRISMA) guidelines. An electronic search of the following databases was undertaken: MEDLINE (from 1946 to December 2019), Embase (from 1974 to December 2019), and Cochrane Library (from 1990 to December 2019). The latest search was performed on December 17, 2019. Different combinations of keywords “Glasgow prognostic score” or “GPS,” “C-reactive protein to albumin ratio” or “CAR,” “C-reactive protein” or “CRP,” “Albumin” or “ALB,” and “sarcoma” were used for the preliminary search. Since OVIDsp (http://ovidsp.dc2.ovid.com/) contains both Embase and MEDLINE databases, we used OVIDsp to search these two databases together. Full search strategy of Embase and MEDLINE using OVIDsp is provided in [Supplementary-material supplementary-material-1], and full search strategy of Cochrane Library is provided in [Supplementary-material supplementary-material-1]. At the same time, relevant studies were also identified by a manual search of references of initially identified articles. Nonhuman study or non-English articles were excluded. Two investigators reviewed the titles and abstracts identified in the search.

### 2.2. Study Inclusion/Exclusion Criteria

Studies were considered eligible if they met all of the following inclusion criteria: (1) studies conducted on patients with sarcoma; (2) studies that investigated the prognostic value of pretreatment GPS or CAR for overall survival (OS), disease-specific survival DSS (DSS), or disease-free survival (DFS; the event-free survival, recurrence-free survival, and progression-free survival are treated as DFS); (3) studies that provided hazard ratios (HRs) along with their 95% CIs (confidence intervals) by either univariate or multivariate survival analysis; and (4) case-control studies, cohort studies, or randomized controlled trials. Studies were excluded based on any of the following exclusion criteria: (1) literature published as case reports, letters, editorials, abstracts, reviews, or expert opinions; (2) studies not based on human patients; and (3) studies focused on tumors that are not within the scope of sarcomas. When the same patient population was involved in two or more studies, only the last or complete study was chosen.

### 2.3. Data Extraction

Eligible publications were reviewed independently by two investigators (E.F. and X.W.). The data extraction was performed by two investigators (E.F. and X.W.). Disagreements were resolved by consulting the senior reviewer (X.Z.). A standardized data collection form was defined previously with the following items: first author, year of publication, study design, country of origin, sample size, histology type, metastasis case numbers, follow-up period, HR estimates with corresponding 95% CIs, and covariates for adjustment. HRs calculated from multivariate analyses were extracted preferentially where available. Otherwise, HRs calculated from univariate analyses were extracted. OS was the primary outcome of interest. DSS and DFS were secondary outcomes.

### 2.4. Assessment of Quality

Two reviewers independently assessed the risk of bias for each study. The Newcastle-Ottawa Quality Assessment Scale (NOS) was applied to assess the qualities of cohort studies. A study with NOS > 7 was regarded as a high-quality study [43]. The tool of ROBINS-I (Risk Of Bias In Nonrandomized Studies of Interventions) was also applied to assess the risk of bias of the included studies [44]. The ROBINS-I tool contains five levels of bias judgment: low, moderate, serious, critical, and no information [44]. Since the GPS and CAR value are both inflammation-based factors, we treat the concomitant conditions (for example, infectious diseases or anti-inflammatory drug treatment) that could possibly affect the system inflammation status of the patients as the most import confounder in the bias assessment by ROBINS-I. Another important confounder is the treatment option received by the patients since different treatment methods might affect the survival outcome. According to the ROBINS-I guidance, the most nonrandomized study will be judged at least at moderate overall risk of bias [44]. Since all the included studies are nonrandomized respective cohort studies, we use a relatively less strict standard and the studies with only critical risk of bias will be excluded in the meta-analysis.

### 2.5. Statistical Analysis

Statistical analysis was carried out using the STATA statistical software package version 15.0 (Stata Corporation, College Station, TX). Combined HRs and Forrest plots were used to estimate the prognostic role of GPS and CAR in sarcoma patients. For studies that reported HRs for GPS 1 and GPS 2 separately, we combined these 2 groups into a single group and calculated a combined HR to analyze the prognostic role of the overall elevated GPS as previously reported [45]. The Cochrane *Q* test (*p* < 0.05 indicated a high level of heterogeneity) and *I*^2^ (values of 25%, 50%, and 75% corresponding to low, moderate, and high degrees of heterogeneity, respectively) were used to evaluate the heterogeneity between eligible studies. When homogeneity was good, a fixed-effects model was used. When heterogeneity was high, a random-effects model was used [46]. An observed HR > 1 indicated worse outcome for an elevated GPS or higher CAR level. Begg's test and Egger's test on the asymmetry of a funnel plot were performed to test any existing publication bias. If the evidence of publication bias was found, a trim and fill method will be adopted to check and revise the combined HRs [47]. If heterogeneity was found between included studies, metaregression analyses or subgroup analyses will be performed to investigate the sources of heterogeneity if available [48]. All statistical tests were two-tailed, and *p* < 0.05 was considered statistically significant.

## 3. Results

### 3.1. Search Results

**Figure 1:**
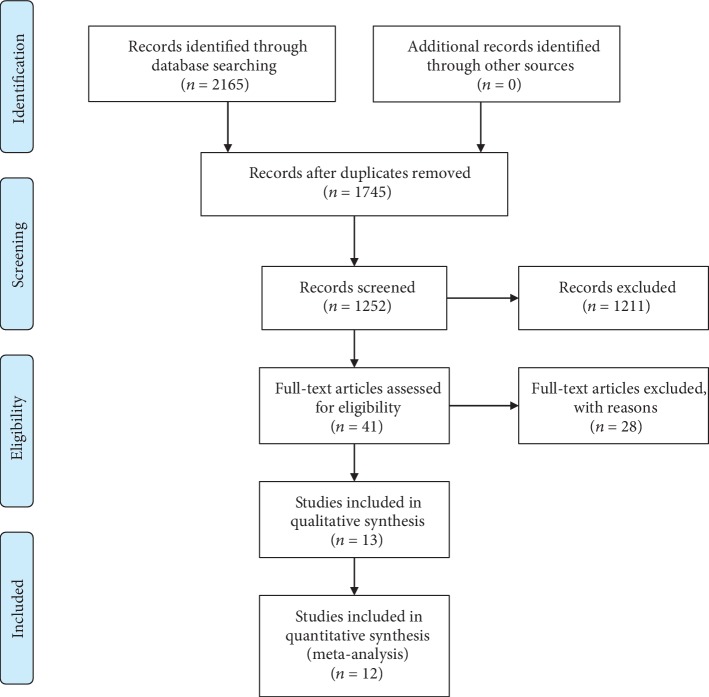
PRISMA flowchart presenting the steps of record search and selection.

The literature search flow chart is shown in Figure 1. Two thousand one hundred and sixty-five records in total were found in the initial search of the three databases, and 420 duplicate articles were deleted after duplicate checking. Nonhuman researches and non-English articles were also removed. One thousand two hundred and fifty-two records were left for title and abstract screening. During the title and abstract screening process, only 6 studies were considered to be with controversy, and the full texts of those controversial records were reviewed to assess the eligibility. Finally, 41 articles (kappa = 0.919, *p* < 0.001) were regarded as potentially relevant articles for a full-text review. Twenty-eight articles were removed due to lack of applicable survival data for the GPS or CAR. While almost all the eligible studies used GPS 0 as the reference group, one study used GPS 2 as the reference group [42]. This exceptional study was excluded because the data cannot be adequately transformed. Finally, 12 eligible studies with 2695 patients were included in this meta-analysis [30–41]. There is no disagreement among the two investigators during the data extraction process.

### 3.2. The Characteristics of the Included Studies

Twelve studies from 2016 to 2019 investigating the prognostic role of the GPS or CAR for sarcoma patients were included, with 2695 patients. The main features of the 12 included studies are summarized in Table 1. In short, four studies were conducted in Europe (1 in UK and 3 in Denmark) [30, 32, 35, 40], while the other eight were in Asia (6 in China and 2 in Japan) [31, 33, 34, 36–41]. The sample size ranged from 83 to 888, while the follow-up time ranged from 20.3 months to 19 years. All the 12 included studies were retrospectively designed. Five studies focused on bone sarcoma patients [30, 31, 33, 34, 41], another five focused on soft tissue sarcoma patients [35–39], and the other two focused on both bone and soft tissue sarcoma patients [32, 40]. Five of the 12 included studies declared that patients with medical conditions known to affect systemic inflammation status were excluded [31, 33, 34, 36, 38]. According to the Newcastle-Ottawa Scale criteria, all 12 articles were rated as high quality (Table 1). However, since all the included studies are nonrandomized cohort studies, six of the studies are judged as at moderate overall risk of bias, and another six are judged as at serious overall risk of bias (Table 2). The main cause of the bias in the included studies was either not appropriately adjusted for concomitant inflammation conditions or not appropriately adjusted for treatment options.

Survival outcomes of the included studies are summarized in Table 3. Eleven studies investigated the prognostic value of GPS for sarcoma patients. Seven of them reported the prognostic role of GPS for OS [30, 31, 33, 34, 36, 38, 39], four of them reported the prognostic role of GPS for DSS [30, 32, 35, 40], and another four reported the prognostic role of GPS for DFS [32, 36–38]. Nine of the eleven studies evaluated traditional GPS, one [32] evaluated mGPS (modified GPS), two [36, 37] evaluated Hs-mGPS (high sensitive modified GPS), and the other one [38] evaluated both traditional GPS and mGPS.

Four studies investigated the prognostic role of CAR for sarcoma patients [33, 34, 36, 41]. All of the four reported the prognostic role of CAR for OS [33, 34, 36, 41], and only two of them reported the prognostic role of CAR for DFS [36, 41]. No study has reported the prognostic role of CAR for DSS.

### 3.3. Prognostic Role of GPS

**Figure 2:**
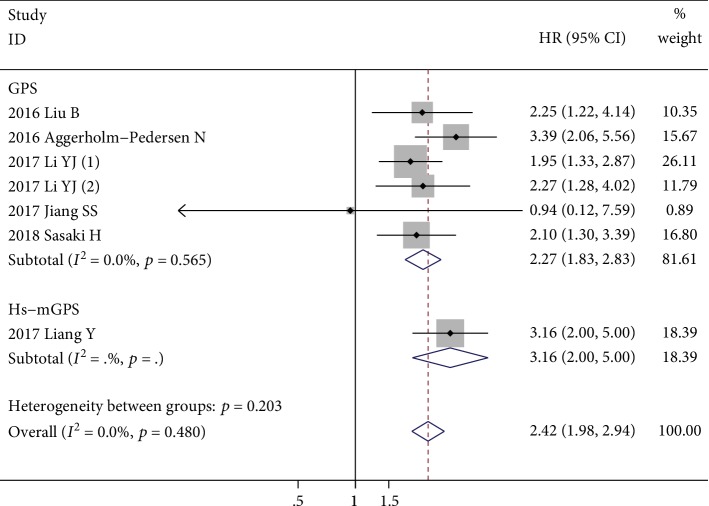
The forest plot about the association between elevated GPS and OS. The pooled effect was calculated using a fixed-effects model.

**Figure 3:**
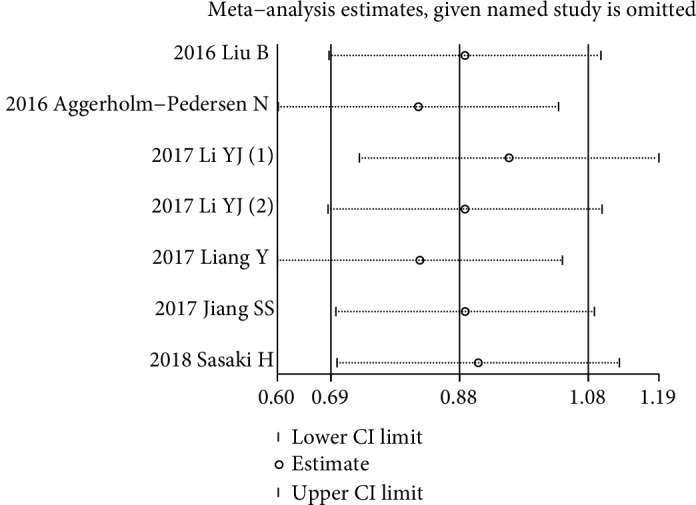
The plot of sensitivity analysis showing the influence of each one of the included study.

Seven studies with 1146 patients investigated the prognostic role of GPS for OS [30, 31, 33, 34, 36, 38, 39], and only two of them reported multivariate-adjusted HRs and 95% CIs [30, 31]. There was no significant heterogeneity among these studies (*I*^2^ = 0.0%, *p* = 0.480). The fixed-effects model revealed that the elevated GPS (both score 1 and score 2) was significantly correlated with poor OS (HR = 2.42; 95% CI: 1.98-2.94; *p* < 0.001) (Figure 2). Sensitivity analysis revealed that the combined HR is stable after excluding each one of the included studies (Figure 3). Subgroup analysis was conducted based on different histology subtypes, multivariate or univariate analysis, patients' ethnicity, inflammation diseases excluded or not, sample sizes, GPS subtype, and metastasis status. The results showed that the elevated GPS was firmly correlated with poor OS among these different subgroups. The details of the subgroup analyses are shown in Table 4.

**Figure 4:**
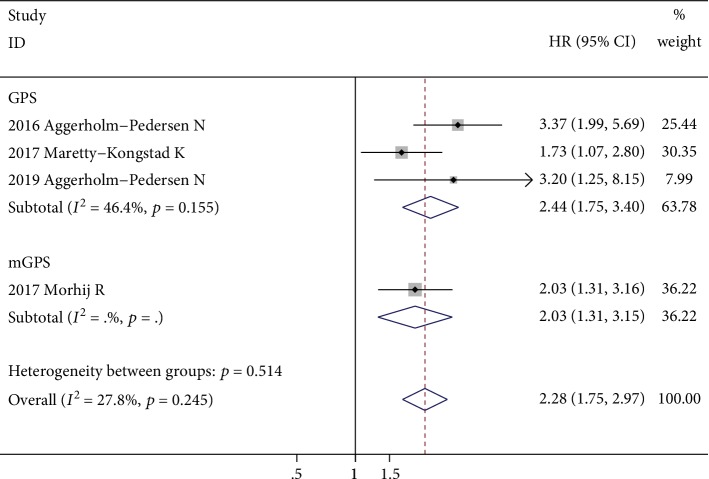
Forest plot showing the association between elevated GPS and DSS in patients with sarcoma. The pooled effect was calculated using a fixed-effects model.

Four studies with 1436 patients investigated the prognostic role of GPS for DSS [30, 32, 35, 40], and all of them reported multivariate-adjusted HRs and 95% CIs. The fixed-effects model revealed that the elevated GPS was significantly correlated with poor DSS (HR = 2.28; 95% CI: 1.75-2.97; *p* < 0.001), with no significant heterogeneity (*I*^2^ = 27.8%, *p* = 0.245) (Figure 4). After excluding the one study which investigated mGPS, the results showed that the elevated GPS was still significantly correlated with poor DSS (HR = 2.44; 95% CI: 1.75-3.40; *p* < 0.001); however, a moderate level of heterogeneity was identified (*I*^2^ = 46.4%%, *p* = 0.155). Since the HRs and their 95% CIs used for combination are all covariate-adjusted, this result suggested that the elevated GPS is an independent prognostic indicator for DSS in patients with sarcoma.

**Figure 5:**
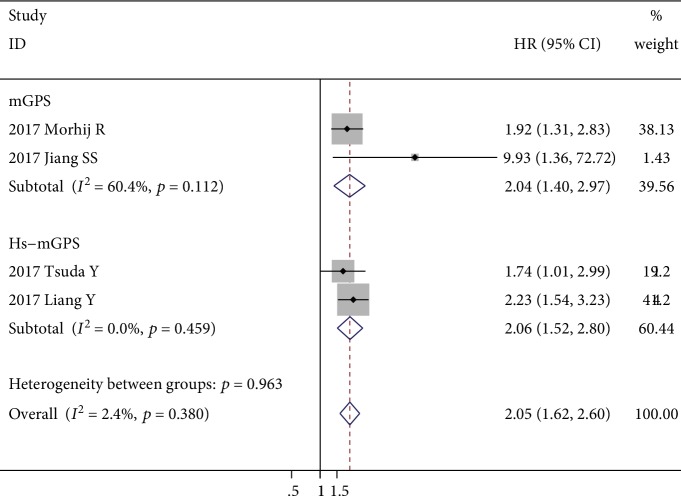
Forest plot showing the association between elevated GPS and DFS in patients with sarcoma. The pooled effect was calculated using a fixed-effects model.

Four studies with 684 patients evaluated the prognostic role of GPS for DFS, and only one of them reported covariate-adjusted HR [32, 36–38]. Two of the included studies evaluated the Hs-mGPS. One study evaluated the mGPS. Another one study evaluated both the GPS and mGPS, and only the survival outcome of the mGPS was included in the meta-analysis. Since no heterogeneity (*I*^2^ = 2.4%, *p* = 0.380) was detected, a fixed-effects model was adopted. The results revealed that the elevated GPS was significantly correlated with poor DFS (HR = 2.05; 95% CI: 1.62-2.60; *p* < 0.001) (Figure 5). Subgroup analysis revealed that both the elevated GPS and mGPS were significantly correlated with poor DFS (HR = 2.04 and HR = 2.06, respectively), although a moderate level of heterogeneity (*I*^2^ = 60.4%, *p* = 0.112) was found among mGPS of the two studies investigated (Figure 5).

### 3.4. Prognostic Role of CAR

**Figure 6:**
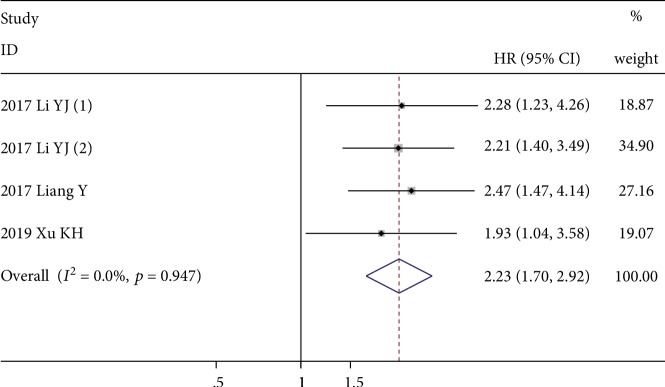
Forest plot showing the association between elevated CAR and OS in patients with sarcoma. The pooled effect was calculated using a fixed-effects model.

Four studies with 627 patients evaluated the prognostic role of CAR for OS, and all of them reported multivariate-adjusted HRs [33, 34, 36, 41]. The fixed-effects model revealed that a higher CAR value was significantly correlated with poor OS (HR = 2.23; 95% CI: 1.70-2.92; *p* < 0.001), with no significant heterogeneity (*I*^2^ = 0.0%, *p* = 0.947) (Figure 6).

**Figure 7:**
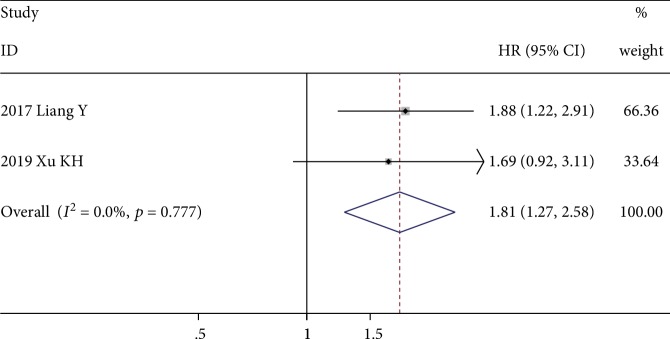
Forest plot showing the association between elevated CAR and DFS in patients with sarcoma. The pooled effect was calculated using a fixed-effects model.

Two studies with 289 patients evaluated the prognostic role of CAR for DFS, and all of them reported multivariate-adjusted HRs [36, 41]. The fixed-effects model revealed that higher a CAR value was significantly correlated with poor DFS (HR = 1.81; 95% CI: 1.7-2.58; *p* = 0.001), with no significant heterogeneity (*I*^2^ = 0.0%, *p* = 0.777) (Figure 7).

### 3.5. Publication Bias

**Figure 8:**
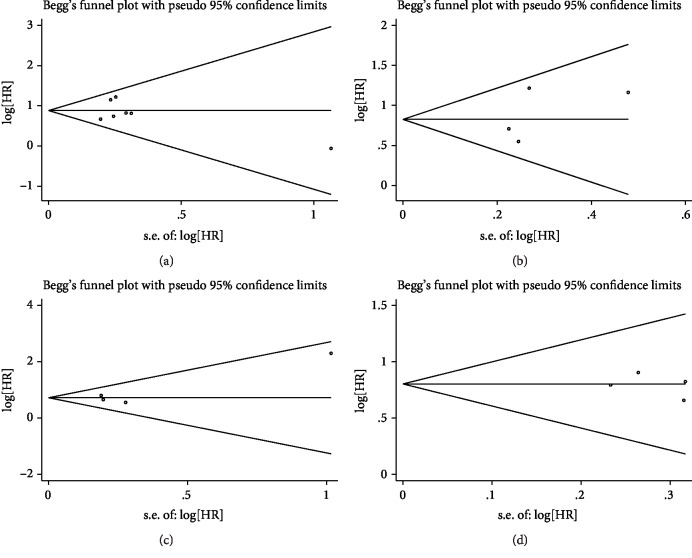
Analysis of publication bias. (a) Begg's funnel plot about the association between GPS and OS. (b) Begg's funnel plot about the association between GPS and DSS. (c) Begg's funnel plot about the association between GPS and DFS. (d) Begg's funnel plot about the association between CAR and OS.

Begg's test and Egger's test both showed no evidence of significant publication bias (*p* = 1.000 and *p* = 0.586, respectively) when evaluating the prognostic role of GPS for OS (Figure 8(a)). Begg's test and Egger's test both showed no evidence of significant publication bias (*p* = 0.734 and *p* = 0.481, respectively) when evaluating the prognostic role of GPS for DSS (Figure 8(b)). Begg's test and Egger's test both showed no evidence of significant publication bias (*p* = 1.000 and *p* = 0.274, respectively) when evaluating the prognostic role of GPS for PFS (Figure 8(c)). Begg's test and Egger's test both showed no evidence of significant publication bias (*p* = 1.000 and *p* = 0.631, respectively) when evaluating the prognostic role of CAR for OS (Figure 8(d)).

## 4. Discussion

Although local condition could be controlled by surgical treatment and adjuvant radiotherapy, about 50% of sarcoma patients with adequate local control develop distant metastases and ultimately die from their disease [4]. Recently, it also has been shown that surgical margins are not predictive of local recurrence and survival in high-grade myxofibrosarcoma [49, 50]. This indicates that the inherent invasive characteristics of the malignancy itself might be more import when it comes to relapse or not. Increasing evidence has revealed that cancer-associated systemic inflammation and malnutrition affect the prognosis of cancer patients [5–8]. Since a high-CRP level and a low-ALB level both have been found to be associated with poor survival in cancer patients [11–14], they might represent an aggressive propensity of the cancer and thus might serve as a factor for risk stratification. More interestingly, a study revealed that the high level of serum CRP was significantly correlated with PD-L1 (programmed death-ligand 1) positivity in patients with non-small-cell lung cancer [13]. Several studies have also discovered that PD-L1 is expressed in about 30–40% of some subtypes of sarcomas [51, 52]. Thus, elevated CRP levels might also represent PD-L1 positivity in malignancies including sarcomas.

The GPS was first described by Forrest et al. [15] and found to have a comparable prognostic value to the conventional combination of stage and performance status in patients with inoperable non-small-cell lung cancer. The traditional GPS is derived by allocating one point each for elevated CRP (>10 mg/L) and hypoalbuminemia (serum albumin < 35 g/L), so that patients with both, one, or none of these conditions would have a score of 2, 1, or 0, respectively [15]. For the modified GPS (mGPS), patients with hypoalbuminemia were assigned a score of 0 in the absence of an elevated C-reactive protein [17, 32, 38]. For the high-sensitivity modified GPS (Hs-mGPS), 3 mg/L (rather than 10 mg/L) is used as the CRP cut-off value [36, 37, 42]. Numerous studies have revealed that an elevated GPS, mGPS, or Hs-mGPS is significantly correlated with poor survival outcome in multiple types of malignancies including sarcoma [17–25, 32, 36–38, 42].

The results of the present study revealed that the elevated GPS is significantly correlated with OS, DSS, and DFS in sarcoma patients. Moreover, the results indicated that the elevated GPS is an independent prognostic factor for DSS since all the HRs used for combination are covariate-adjusted. It has to be noted that several of the included studies have chosen the mGPS or Hs-mGPS as the investigated parameters [32, 36–38]. However, it does not seem to matter which of the three GPS subtypes was used since no heterogeneity was detected among the included studies. In evaluating the prognostic role of GPS for PFS, two studies used mGPS and another two used Hs-mGPS; the results of the meta-analysis still revealed that the elevated GPS was significantly correlated with poor DFS (HR = 2.05; 95% CI: 1.62-2.60; *p* < 0.001) with no significant heterogeneity. These results suggest that the GPS might be a promising factor that could be used for risk stratification. It also has to be mentioned that one study [38] has investigated both the GPS and mGPS and came into a negative conclusion for the GPS in the multivariate analysis model. However, this study also has revealed that the elevated GPS or mGPS is significantly correlated with poor OS or PFS in the univariate analysis model. We think that one reason responsible for this discrepancy is that this study used the GPS and mGPS as a covariate for each other in the multivariate model.

The CAR is simply the ratio of the CRP level to the ALB level. The results of the present study also revealed that a higher CAR is significantly correlated with OS and DFS for sarcoma patients. The Higher CAR is an independent prognostic factor for OS and DFS since all the HRs used for combination are covariate-adjusted. However, it has to be noted that the cut-off value for the CAR varies among the 4 included studies, ranging from 0.1035 to 1.5. The inconsistency of the cut-off values holds back the clinical use of the CAR as a convenient indicator at least before the optimal cut-off value is confirmed.

There are some limitations in the present study as follows.

First, this study is not yet successfully registered online. Actually, we have submitted the registration of this study on PROSPERO at the beginning of this study; however, the registration record is still being assessed by the editorial team when this manuscript is submitted. Nevertheless, to our knowledge, this meta-analysis is the first to evaluate the prognostic role of GPS or CAR for sarcoma. Our systemic literature search identified no systemic review or meta-analysis on this subject, either for the GPS or for the CAR.

Second, only a small number of studies were included in this meta-analysis, and all of them were nonrandomized retrospective cohort studies. The ROBINS-I bias assessments revealed that six of the included studies were regarded as with serious overall risk of bias. Only six of the included studies reported that patients with concomitant inflammatory conditions were excluded from their study. Since the GPS and CAR value are both inflammation-based factors, it would be better to exclude those patients with concomitant inflammatory conditions. Different treatment options may also affect the survival outcome of the patients. For example, one study reported that the baseline CRP level did not predict poor clinical outcome in STS patients receiving neoadjuvant radiotherapy [53]. Neoadjuvant radiotherapy may have improved the outcome of those STS patients with an elevated CRP level; so on the contrary, patients with elevated CRP at diagnosis may be good candidates for neoadjuvant radiotherapy [53]. The GPS may also serve a similar function as the CRP, so it would be better for the studies to perform the survival analysis adjusted for different treatment methods received by the patients.

Third, sarcomas represent a heterogeneous group of tumors including multiple subtypes probably with different status of inflammation and malnutrition. It would be better to analyze sarcoma based on different histology subtypes. Since most of the included studies involved multiple histology subtypes, we could not conduct subgroup analyses for each of the histology subtypes.

Fourth, several of the included studies reported the modified GPS instead of the traditional GPS. Although no severe heterogeneity was detected among the included studies, it would be better for future studies to find out which one of the GPS subtypes is optimal to be used as a risk factor for sarcoma.

Finally, although no significant heterogeneity was detected when conducting the overall meta-analysis, some degree of heterogeneity was still identified on the subgroup analyses. In fact, the different histology subtypes of the sarcoma and different GPS subtype all suggest an inherent heterogeneity among the included studies. The metastasis status of the tumor may also contribute to the heterogeneity. A previous study also found that higher proportion of metastatic STS patients had an elevated serum CRP level than that of patients without metastasis [14, 21]. Due to a small number of included studies, we could not perform specific subgroup analysis based on different histology subtypes, different metastatic status, and different GPS subtypes.

Considering the above-mentioned drawbacks, we recommend future studies to focus on a specific histology subtype of sarcoma, specific type of GPS, different metastatic status of the tumor, and different treatment methods received by the patients if available. Well-designed large-scale prospective researches are warranted to confirm the independent prognostic role of GPS for the survival of sarcoma patients. Fundamental researches are also required to elucidate the hidden mechanism of sarcoma-associated inflammation responses and malnutrition.

## 5. Conclusions

The present study revealed that both the GPS and CAR are predictive of survival in patients with sarcoma. The elevated GPS is an independent prognostic factor for DSS in sarcoma patients and might serve as a factor for risk stratification. Although CAR is also predictive of survival for sarcoma patients, the inconsistency and uncertainty of its cut-off values hold back its use in clinical practice at least before the optimal cut-off value is confirmed.

## Figures and Tables

**Table 1:** Basic characteristics of the included studies.

Study (year)	Country	Study design	Sample size (*n*)	Metastasis case (*n*)	Histology	Histology subtypes	Follow-up period	Inflammation	NOS scores
Aggerholm-Pedersen et al. (2016) [30]	Denmark	Rs	172	0	BS	Chondrosarcoma (*n* = 63), Ewing/osteosarcoma (*n* = 109)	Median: 8.8 y Range: 4.3-19 y	NA	8

Liu et al. (2016) [31]	China	Rs	162	19	BS	Osteosarcoma (*n* = 162)	Median:28.2 m Range: 3.1-124.1 m	Excluded	8

Morhij et al. (2017) [32]	UK	Rs	111	0	STS/BS	Soft tissue sarcoma (*n* = 69), bone sarcoma (42)	Median:50 m Range: 34-78 m	NA	8

Li et al. (1) (2017) [34]	China	Rs	216	32	BS	Osteosarcoma (*n* = 216)	Median:31.5 m	Excluded	9

Li et al. (2) (2017) [33]	China	Rs	122	17	BS	Ewing's sarcomas (*n* = 122)	Median:35 m	Excluded	9

Maretty-Kongstad et al. (2017) [35]	Denmark	Rs	888	0	STS	Liposarcoma, UPS, leiomyosarcoma, dermatofibrosarcoma, synovial sarcoma, MPNST, and others	Mean: 5.7 y Range: 0.1-22 y	NA	7

Liang et al. (2017) [36]	China	Rs	206	NA	STS	MFH (*n* = 56), fibrosarcoma (*n* = 38), synovial sarcoma (*n* = 25), liposarcoma (*n* = 22), leiomyosarcoma (*n* = 9), rhabdomyosarcoma (*n* = 13), ASPS (*n* = 8), angiosarcoma (*n* = 6), MPNST (*n* = 8), mesenchymal chondrosarcoma (*n* = 7), others (*n* = 14)	Median:75.5 m Range: 8-136 m	Excluded	9

Tsuda et al. (2017) [37]	Japan	Rs	202	0	STS	UPS (*n* = 74), myofibrosaroma (*n* = 46), leiomyosarcoma (*n* = 19), dedifferentiated liposarcoma (*n* = 11), fibrosarcoma (*n* = 9), synovial sarcomas (*n* = 5), pleomorphic liposarcoma (*n* = 5), others (*n* = 33)	Mean: 58 m 95% CI: 52-62 m	NA	7

Jiang et al. (2017) [38]	China	Rs	165	97	STS	Fibrohistiocytic tumor (*n* = 41), undifferentiated sarcoma (*n* = 93), smooth muscle tumor (*n* = 10), skeletal muscle tumor (*n* = 17), adipocytic tumor (*n* = 3), fibroblastic/myofibroblastic tumor (*n* = 1)	Mean: 73.7 m Range: 17.2-533.8 m	Excluded	8

Sasaki et al. (2018) [39]	Japan	Rs	103	NA	STS	Soft tissue spindle cell sarcomas (*n* = 103)	60.6 ± 39.6 m	NA	7

Aggerholm-Pedersen et al. (2019) [40]	Denmark	Rs	265	265	STS/BS	STS (*n* = 202): UPS (*n* = 41), leiomyosarcoma (n = 31), liposarcoma (*n* = 27), synovial sarcoma (*n* = 27), MPNST (*n* = 15), others (*n* = 63) BS (*n* = 63): osteosarcoma (*n* = 28), Ewing's sarcoma (*n* = 8), chondrosarcoma (*n* = 19), others (*n* = 8)	Median: 0.9 y	NA	8

Xu et al. (2019) [41]	China	Rs	83	9	BS	Ewing's sarcomas (*n* = 83)	Mean: 20.3 m Range: 1-84 m	Excluded	8

Rs: retrospective study; STS: soft tissue sarcoma; BS: bone sarcoma; m: month; y: year; NA: not available; UPS: undifferentiated pleomorphic sarcoma; MPNST: malignant peripheral nerve sheath tumor; MFH: malignant fibrous histiocytoma; ASPS; alveolar soft part sarcoma.

**Table 2:** Risk of bias assessment according to ROBINS-I.

Study	Bias due to confounding	Bias in selection of participants	Bias in classification of interventions	Bias due to deviations from intended interventions	Bias due to missing data	Bias in measurement of outcomes	Bias in selection of the reported result	Overall bias
Aggerholm-Pedersen et al. (2016)	Serious	Low	Low	Low	Moderate	Low	Low	Serious
Liu et al. (2016)	Moderate	Low	Low	Low	Serious	Low	Low	Serious
Morhij et al. (2017)	Serious	Low	Low	Low	Low	Low	Low	Serious
Li YJ et al. (1) (2017)	Low	Low	Low	Low	Low	Low	Moderate	Moderate
Li YJ et al. (2) (2017)	Low	Low	Low	Low	Low	Low	Moderate	Moderate
Maretty-Kongstad et al. (2017)	Serious	Low	Low	Low	Moderate	Low	Low	Serious
Liang et al. (2017)	Low	Low	Low	Low	Low	Low	Moderate	Moderate
Tsuda et al.(2017)	Moderate	Low	Low	Low	Moderate	Low	Moderate	Moderate
Jiang et al. (2017)	Moderate	Low	Low	Low	Low	Low	Low	Moderate
Sasaki et al. (2018)	Serious	Low	Low	Low	Low	Low	Serious	Serious
Aggerholm-Pedersen et al. (2019)	Serious	Low	Low	Low	Low	Low	Low	Serious
Xu et al. (2019)	Moderate	Low	Low	Low	Low	Low	Low	Moderate

ROBINS-I is short for Risk Of Bias In Nonrandomized Studies of Interventions, which contains five levels of bias judgment: low, moderate, serious, critical, and no information.

**Table 3:** Survival analysis data of the included studies.

Study	Marker	Cut-off	Survival analysis	HR	95% CI	*p* value	Variables for multivariate analysis
Aggerholm-Pedersen et al. [30]	GPS	—	DSS^a^	3.367	1.993-5.688	<0.001	Age, tumor size, histology, margin, soft tissue extension, comorbidity
			OS^a^	3.387	2.062-5.564	<0.001	
Liu et al. [31]	GPS	—	OS	2.25	1.222-4.145	0.009	Enneking stage, metastasis, tumor site, CRP, NLR, PLR, LMR
Morhij et al. [32]	mGPS	—	CSS	2.03	1.31-3.16	0.002	Tumor size, grade, CRP, ALB, WCC
			RFS	1.92	1.31-2.83	0.001	Tumor size, grade, CRP, ALB
Li et al. (1) [34]	GPS	—	OS	1.95	1.33-2.87	0.001	Univariate analysis
	CAR	0.210	OS	2.62	1.70-4.03	<0.001	Metastasis, tumor site
Li et al. (2) [33]	GPS	—	OS	2.27	1.28-4.02	0.006	Univariate analysis
	CAR	0.225	OS	2.28	1.23-4.26	0.009	Enneking stage, tumor site
Maretty-Kongstad et al. [35]	GPS	—	DSS^a^	1.731	1.070-2.801	0.026	Age, tumor size, grade, histology, tumor depth, comorbidity
Liang et al. [36]	Hs-mGPS	—	OS^a^	3.162	2.000-4.998	<0.001	Univariate analysis
			DFS^a^	2.232	1.542-3.231	<0.001	
	CAR	0.1035	OS	2.47	1.47-4.14	0.001	Grade
			DFS	1.88	1.22-2.91	0.004	Age, grade
Tsuda et al. [37]	Hs-mGPS	—	EFS	1.74	1.01-2.99	0.046	Age, sex, UICC stage, margin, ECOG PS
Jiang et al. [38]	GPS	—	OS	0.941	0.117-7.585	0.954	mGPS, age, pathological grade, primary tumor depth
			PFS	0.312	0.047-2.664	0.353	
	mGPS		OS	1.660	0.22-12.534	0.623	GPS, age, pathological grade, primary tumor depth
			PFS	9.932	1.357-72.716	0.024	
Sasaki et al. [39]	GPS	—	OS	2.098	1.229-3.388	0.002	Univariate analysis
Aggerholm-Pedersen et al. [40]	GPS	—	DSS^a^	3.195	1.253-8.147	0.015	Age, comorbidity, histology, site of metastasis
Xu et al. [41]	CAR	1.5	OS	1.930	1.040-3.579	0.037	Age, Frankel score, resection mode, D-dimer, PLR
			DFS	1.687	0.916-3.106	0.093	Age, Frankel score, metastasis, resection mode, D-dimer, PLR

GPS: Glasgow prognostic score; mGPS: modified GPS; Hs-mGPS: high sensitive modified GPS; CAR: C-reactive protein to albumin ratio; OS: overall survival; DSS: disease-specific survival; DFS: disease-free survival; RFS: recurrence-free survival; EFS: event-free survival; PFS: progression-free survival; HR: hazard ratio; CI: confidence interval; CRP: C-reactive protein; NLR: neutrophil to lymphocyte ratio; PLR: platelet to lymphocyte ratio; LMR: lymphocyte to monocyte ratio; ALB: albumin; WCC: white cell counts; UICC: Union for International Cancer Control; ECOG PS: Eastern Cooperative Oncology Group performance status.^a^These studies reported HRs for GPS 1 and GPS 2 separately. We combined these 2 groups and calculated a combined HR for the overall elevated GPS.

**Table 4:** Subgroup analyses of the prognostic role of GPS for OS.

Subgroup	No. of studies	No. of participants	HR	95% CI	*p* value	*I* ^2^
Histology type						
Bone sarcoma	4	672	2.35	1.84-3.01	*p* < 0.001	1.0%
Soft tissue sarcoma	3	474	2.53	1.83-3.52	*p* < 0.001	15.01%
Analysis method						
Multivariate analysis	3	449	2.78	1.90-4.05	*p* < 0.001	5.2%
Univariate analysis	4	647	2.29	1.82-2.89	*p* < 0.001	0.0%
Ethnicity						
Asian	6	984	2.27	1.83-2.81	*p* < 0.001	0.0%
Other	1	162	3.39	2.06-5.56	*p* < 0.001	—
Inflammation diseases						
Excluded	5	881	2.31	1.82-2.94	*p* < 0.001	0.0%
Not mention	2	265	2.64	1.87-3.73	*p* < 0.001	46.2%
Sample size						
*n* < 200	5	724	2.27	1.80-2.88	*p* < 0.001	0.0%
*n* > 200	2	422	2.78	1.94-3.97	*p* < 0.001	0.0%
GPS subtype						
GPS	6	940	2.27	1.83-2.83	*p* < 0.001	0.0%
Hs-mGPS	1	206	3.16	2.00-5.00	*p* < 0.001	—
Metastasis status						
With metastatic cases	6	984	2.27	1.83-2.81	*p* < 0.001	0.0%
Without metastatic cases	1	162	3.39	2.06-5.57	*p* < 0.001	—

## Data Availability

The data supporting this study are from previously reported articles, which have been cited as references. The processed data are available in Table 3 of this article.

## Supplementary Materials

Supplementary Materials PRISMA 2009 checklist. Appendix A: full search strategy of Embase and MEDLINE using OVIDsp. Appendix B: full search strategy of Cochrane Library.
