# Supplementary material for: The Prognostic Role of Glasgow Prognostic Score and C-reactive Protein to Albumin Ratio for Sarcoma: A System Review and Meta-Analysis
Source: Dis Markers. 2020 Jan 7;2020:8736509. doi: 10.1155/2020/8736509 (PMC6969993; doi:10.1155/2020/8736509)
Supplement: Supplementary Materials — PRISMA 2009 checklist. Appendix A: full search strategy of Embase and MEDLINE using OVIDsp. Appendix B: full search strategy of Cochrane Library. [file 8736509.f1.zip › new supplementa 1.pdf]

Supplementary Materials

PRISMA 2009 checklist.
Appendix A: Full search strategy of Embase and MEDLINE using OVIDsp.
Appendix B: Full search strategy of Cochrane library.

Appendix A: Full search strategy of Embase and MEDLINE using OVIDsp.

#	Searches (Keyword)	Results	Search Type
1	(Glasgow prognostic score and sarcoma).mp. [mp=ti, ab, hw, tn, ot, dm, mf, dv, kw, fx, dq, nm, kf, ox, px, rx, ui, sy]	27	Advanced
2	(GPS and sarcoma).mp. [mp=ti, ab, hw, tn, ot, dm, mf, dv, kw, fx, dq, nm, kf, ox, px, rx, ui, sy]	36	Advanced
3	(C-reactive protein to albumin ratio and sarcoma).mp. [mp=ti, ab, hw, tn, ot, dm, mf, dv, kw, fx, dq, nm, kf, ox, px, rx, ui, sy]	6	Advanced
4	(CAR and sarcoma).mp. [mp=ti, ab, hw, tn, ot, dm, mf, dv, kw, fx, dq, nm, kf, ox, px, rx, ui, sy]	263	Advanced
5	(C-reactive protein and sarcoma).mp. [mp=ti, ab, hw, tn, ot, dm, mf, dv, kw, fx, dq, nm, kf, ox, px, rx, ui, sy]	635	Advanced
6	(CRP and sarcoma).mp. [mp=ti, ab, hw, tn, ot, dm, mf, dv, kw, fx, dq, nm, kf, ox, px, rx, an, ui, sy]	234	Advanced
7	(albumin and sarcoma).mp. [mp=ti, ab, hw, tn, ot, dm, mf, dv, kw, fx, dq, nm, kf, ox, px, rx, ui, sy]	1228	Advanced
8	(ALB and sarcoma).mp. [mp=ti, ab, hw, tn, ot, dm, mf, dv, kw, fx, dq, nm, kf, ox, px, rx, an, ui, sy]	23	Advanced
9	1 or 2 or 3 or 4 or 5 or 6 or 7 or 8	2151	Advanced
10	remove duplicates from 9	1731	Advanced
11	limit 10 to english language	1644	Advanced
12	limit 11 to human	1238	Advanced

The latest search was performed on December 17, 2019.

Appendix B: Full search strategy of Cochrane library.

#	Searches (Keyword)	Results	Search Type
1	Glasgow prognostic score and sarcoma	0	Advanced Search
2	GPS and sarcoma	0	Advanced Search
3	C-reactive protein to albumin ratio and sarcoma	0	Advanced Search
4	CAR and sarcoma	0	Advanced Search
5	C-reactive protein and sarcoma	4	Advanced Search
6	CRP and sarcoma	0	Advanced Search
7	albumin and sarcoma	10	Advanced Search
8	ALB and sarcoma	0	Advanced Search
9	1 or 2 or 3 or 4 or 5 or 6 or 7 or 8	14	Advanced Search
